# ‘Teachers are the guinea pigs’: teacher perspectives on a sudden reopening of schools during the COVID-19 pandemic

**DOI:** 10.1007/s13384-022-00577-6

**Published:** 2023-02-13

**Authors:** Jillian Ryan, Nicole Koehler, Travis Cruickshank, Shane L. Rogers, Mandy Stanley

**Affiliations:** 1 School of Medical and Health Sciences, Edith Cowan University, Perth, WA Australia; 2 Centre for Precision Health, Edith Cowan University, Perth, WA Australia; 3 School of Arts and Humanities, Edith Cowan University, Perth, WA Australia

**Keywords:** COVID-19, Pandemic, Primary Education, Secondary Education, Schoolteachers, Wellbeing, Government Policy

## Abstract

Primary and secondary education systems experienced substantial disruption during the COVID-19 pandemic. However, little is known about how public health policy has affected Australian teachers during the pandemic. This study examines teacher perspectives on a sudden change of policy, whereby schools were abruptly opened to students at the beginning of the pandemic. At the same time, strict social distancing rules applied to the remainder of the population. Qualitative data from 372 Western Australian schoolteachers were analysed using thematic analysis. Results highlight substantial impacts on teachers’ workloads and adverse effects on wellbeing. Perceptions that they were acting as guinea pigs and subjected to different social distancing rules than other citizens were particular stressors. Findings highlight substantial consequences of public health policies on the roles and wellbeing of teachers.

## Introduction

COVID-19 is a severe and infectious respiratory illness that, as of March 2022, has infected more than 350 million individuals worldwide and taken the lives of at least 5.5 million (World Health Organization, 2022). As much as the pandemic has been a public health crisis, it has also caused social and economic crises that have had detrimental effects on the wellbeing of children (Bessell, 2021) and adults (Brindal et al., 2021).

Schools and the broader education sector were severely impacted by the COVID-19 pandemic (Ozamiz-Etxebarria et al., 2021). It is estimated that schools across 185 countries, covering nearly 90% of the world’s student population, closed their doors during the pandemic, many for long periods of 40 + weeks (UNESCO, 2022). These closures forced schools to pivot between face-to-face and remote modes of teaching, particularly in 2020 and 2021, which was enormously disruptive to education, students, teachers, and parents (Pokhrel & Chhetri, 2021). School closures disrupt multiple aspects of home life including the security of childcare, meal provision, and ultimately, learning. Furthermore, closures require schools to rapidly implement remote lesson delivery, which may include combinations of online-delivered lessons and take-home packets, for example; a major change in delivery mode that teachers had to adjust to rapidly (Edmunds, 2020; Pokhrel & Chhetri, 2021). This caused an increase in workload for teachers and likely introduced a new source of occupational stress. Schools systems also suffered from the impacts of broader social and economic effects of the pandemic, including increased economic and family stress, worsening mental health of students and staff, disease outbreaks, and impediments to learning and social development, particularly in special needs educational settings (Hoofman & Secord, 2021; Sharma et al., 2022).

There is limited evidence regarding the impacts of COVID-19 on schoolteachers; however, in 2022, more evidence is emerging. Early research was summarised in a review of eight studies that evaluated teachers’ mental health during the pandemic (Ozamiz-Etxebarria et al., 2021). Findings indicated elevated anxiety (17%), depressive (19%), and stress (30%) symptoms in teachers, with primary stressors relating to emergency switch to online or e-learning, work overload, and uncertainty about the future status of schools (Ozamiz-Etxebarria et al., 2021). A handful of other studies have explored the specific impacts of online or remote teaching on teachers in Indonesia (*N* = 6; Purwanto et al., 2020) and Turkey (*N* = 16; Hebebci et al., 2020), identifying positive and negative perceptions of working from home (Hebebci et al., 2020; Purwanto et al., 2020). More recently, a study with schoolteachers in New South Wales, Australia, similarly identified a range of detrimental impacts that the pandemic had on respondents’ confidence, morale, and professional identities; with many teachers reporting feeling unappreciated during the pandemic (Fray et al., 2022). Another study highlights some of the supporting factors, such as flexibility and supporting uptake of new technologies that enabled teachers to maintain student engagement and protect against the challenges of online learning (Sharma et al., 2022).

It is important to recognise that any potential impacts of the pandemic on schoolteachers are occurring against a backdrop of already-elevated levels of occupational stress among teachers (García-Carmona et al., 2019). Teaching can be a rewarding profession while simultaneously associated with high job demands, levels of emotional labour (e.g. Hagenauer & Volet, 2014), and significant external pressures from stakeholders (e.g. Skourdoumbis, 2014). As a result, research has reported high levels of stress, burnout, and attrition among teachers (Macdonald, 1999; McGrath-Champ et al., 2018). Consistent with this, the most recent data from Safe Work Australia indicate that teachers are the second-highest profession for making mental health compensation claims, following first responders and emergency services (Safe Work Australia, 2015). Many teachers report a desire to leave the profession within the next five years, particularly early-career teachers who have completed less than 10 ten years’ service (Arnup & Bowles, 2016).

A contributing factor to high levels of stress and attrition among teachers is that job demands have increased steadily over recent decades as part of a shift towards mass-education models (Eacute & Esteve, 2000; Skaalvik & Skaalvik, 2018). This has been linked to declining morale as teachers face increasing responsibilities and pressure from growing numbers of internal and external stakeholders, including distal evaluations of success (e.g. national testing schools and school rankings; Eacute & Esteve, 2000). As a result, teachers report feeling underappreciated and undervalued by school families, the public, and in media coverage (Heffernan et al., 2022; Shine & Rogers, 2021).Paradoxically, there is also evidence that teachers are viewed more positively by their communities than teachers perceive (Heffernan et al., 2019). Given the challenging nature of the teaching profession and the severe impacts of the COVID-19 pandemic on teachers and schools, further research that seeks to unpack how this health disaster has impacted teachers is required.

### Study context and objectives

The current study analysed qualitative data collected from Western Australian teachers in the two days following an announcement on Friday, April 17, from the Western Australian Premier that schools would reopen again for the commencement of the second term on the 29th of April 2020 (Laschon, 2020). This announcement came during severe COVID-19 social distancing and movement restrictions. Citizens were instructed to avoid associating with more than two people at a time and keep a 1.5 m physical distance from others wherever possible. The announcement to reopen schools was met with opposition from the education community (The State School Teachers’ Union of W.A. (Inc.), 2020) because it was unclear how schools would implement social distancing policies or whether sufficient personal protective equipment (PPE) and cleaning supplies were available in schools.

Many families decided to keep their children at home, a decision that was actively promoted by the State School Teachers’ Union of WA (2020). Some expressed concerns that it would not be possible to comply with social distancing rules in the classroom, placing teachers, students, and other staff at increased risk of COVID-19 infection. At the time, there was limited evidence available about how COVID-19 was transmitted via children. It was believed that children infrequently contracted or transmitted COVID-19 compared to older children and adults due to the low prevalence of COVID-19 in children (Coffin & Rubin, 2021). However, this assumption was later overturned as it was found that while children did experience less severe disease, they were more likely to transmit COVID-19 to others, primarily due to behavioural factors (e.g. unable to adhere to mask and social distancing rules) and delayed approval of vaccines for children (Coffin & Rubin, 2021; Paul et al., 2021).

The present study explored schoolteachers’ reactions to the Western Australian Premier’s announcement, providing an opportunity to examine how a sudden policy change that was part of the pandemic response strategy impacted teachers as one of the most vulnerable and critical professional groups in our society. The study aimed to investigate teachers’ perspectives on the impacts of COVID-19 on teachers, coping strategies, and feelings about the government’s response to COVID-19.

## Method

Data discussed in this paper were collected as part of a broader cross-sectional survey exploring the impacts of COVID-19 lockdowns on Australians’ mental health, physical health, and social relationships (*N* = 1599; see Rogers & Cruickshank, 2021). The current study includes responses from teachers, educators, and principals of that sample (*n* = 372). Ethical approval was obtained from Edith Cowan University’s Human Research Ethics Committee (reference number 2020-01305-ROGERS). We used an exploratory observational, inductive approach to the study design and data analysis, meaning that the study was not guided by pre-specified hypotheses or theoretical frameworks.

### Materials

Participants completed an anonymous online survey administered via online survey software, Qualtrics, between 17 and 18 April 2020. The survey included three items that collected information about participants’ gender, age (in categories of 5 years), and occupation with response options including full time, part time, or casual. Finally, participants were asked whether they had lost employment or experienced a reduction in hours due to COVID-19.

This study analysed qualitative data obtained via a single-item open-ended survey question that asked participants to describe ‘…how you are currently feeling because of COVID-19, the impacts it has had on you (positive and negative), strategies you are using to cope (if there has been any impact on you), and feelings about the government response’.

### Participants and procedure

Eligible participants included survey respondents who resided in Western Australia; identified themselves as working within the kindergarten to Year 12 educational sector (e.g. teacher, educational assistant, principal); completed the survey on the 17th or 18th of April 2020 (i.e. proximal to the Western Australian premier’s announcement that schools will open); and completed the qualitative portion of the survey.

Participants were recruited via a multi-strategy and low-cost social marketing campaign. A weblink to the online survey was disseminated primarily via social media channels like Facebook and Twitter and the authors’ social and professional networks. Respondents could click the online link and complete the survey on any internet-enabled device. No incentives or reimbursement were offered to participants.

### Data analysis

Qualitative data were analysed using inductive thematic analysis (Braun & Clarke, 2006, 2021). Inductive thematic analysis involves a data-driven and ‘bottom-up’ process of identifying themes within the data; hence, resultant themes are closely tied to the data itself rather than aiming to fit a pre-specified theoretical or conceptual framework. Briefly, the process of thematic analysis consisted of iterative analysis of the data by two authors (NK and JR) to identify themes within the data. This started with an initial reading of the data (familiarisation) and open coding of possible themes or codes reflecting the content of each response. Following this, one author brought together like codes to create thematic categories reflective of participants’ comments. A second author (JR) independently reviewed the themes and how well they fit the data, refining the themes to arrive at the final thematic map.

## Results

A total of 372 participants met the inclusion criteria for the analyses reported here (see Table 1). Most participants were female (*n* = 364) and aged between 41 and 50 years old (*n* = 115). Most participants were in a relationship (*n* = 308) and were employed on a full-time basis (*n* = 260). Less than 8% of participants reported a loss of employment due to COVID-19 (*n* = 10) or a reduction of hours (*n* = 17). Most participants reported that their employment was not affected by COVID-19 (*n* = 345).

**Table 1:** Participant characteristics of West Australian teachers (*N* = 372) surveyed during the COVID-19 pandemic

Age bracket (years)	*n*	Percentage
18–30	44	11.8%
31–40	105	28.2%
41–50	115	30.9%
51–60	91	24.5%
61–70	17	4.6%
*Female or male*		
Female	364	97.8%
Male	7	1.9%
I prefer not to say	1	< 1%
*Employment status*		
Full time	260	70.0%
Part time	96	25.8%
Casual	16	4.3%
*Impact of COVID-19 on employment status*		
Unaffected	345	92.7%
Loss of employment	10	2.7%
Reduced employment	17	4.6%

### Themes in teacher’s reactions to the sudden reopening of schools

Participants provided qualitative responses that ranged from four to 560 words in length (*n* = 372; *M* = 95.2; *SD* = 84.6). Analyses identified four main themes: (1) policy implementation, (2) teacher wellbeing, (3) disease outbreaks, and (4) working conditions. Themes are discussed in detail below.

### Policy implementation

Policy implementation approaches during the pandemic was a focal theme among teachers, particularly as it related to school closures and re-openings. Participants expressed anger, frustration, and distress related to the sudden reopening of schools and the limited time provided to switch between remote and in-person teaching delivery modes or to prepare multiple delivery modes simultaneously. This was seen to fall outside teachers’ capacity since lessons were typically planned well in advance and took days or weeks to prepare, much longer than the changes allowed. One teacher commented:

As a teacher, my biggest stress is work and not knowing what we are meant to do and how to cope with the daily changes we are getting thrown at us. It usually takes a few days to a week to plan half a term’s work, and we are being thrown into it and expected to teach n a different medium without any time to prep, and then after spending quite a few late nights trying to prep for online next term, they tell us that it was all for nothing… (Participant Identification (PID) 623)

Participants also commented on the double standards reflected in policy. Teachers and schools noted that they were expected to follow different rules (e.g. higher density requirements) from the rest of society. This was seen as unfair. One teacher commented:

It is very hard to come to terms with the fact that the government says we cannot sit and have a meal with a couple of friends, but we can spend all day in a room with 30 kids, where social distancing isn’t possible and essentially kids really do not understand how to social distance. (PID 304)

Participants also felt that rapid shifts in school opening status signified disrespect towards teachers and a gross misunderstanding of teachers’ roles, including portraying teachers as babysitters. This sentiment was further reinforced by some participants who felt that economic, rather than educational, drivers were keeping schools open. Several participants used analogies to scapegoats or guinea pigs to explain how they handled this policy shift had treated them.

The government response/attitude to educational settings is extremely unsettling. The impact of having extremely vague directions plus having worked incredibly hard to upskill ourselves and change the mode of teaching to online (in the space of weeks, if not days) AND making sure the learning experiences are available for EVERYONE (including the students who do not have access to the internet thus need hard copies of everything) is phenomenal. I am feeling like a scapegoat / sacrificial lamb/ babysitter, even though, as a whole, teachers have pulled together and worked harder than ever before*.* (PID 628)

I am feeling, as are many of my colleagues, like scapegoats for a human CV19 transmission experiment at the expense of supporting our economy. (PID 52)

A small number of participants made positive comments that praised the public health response to the pandemic or showed appreciation for the task’s difficulty.

I think the government is doing the best job they can, because what is happening is unprecedented so everything they are trying has no proven track record. (PID 62)

### Teacher wellbeing

The second theme captured the pandemics’ impact on teachers’ physical, mental, and financial wellbeing and that of teachers' family, peers, students, and the broader community. Several participants reflected upon how the pandemic had decreased their interest in the teaching profession or mentioned resigning.

I loved teaching but now I’ve lost my passion/spark for it. (PID 49)

Given the potential covid carer’s leave being capped at 20 days, no income or end date in sight, an offsite principal that encourages non-working parents to send kids to school, and being unable to check off OHS requirements to work from home (e.g. noise levels and dedicated work time chunks with my three young children home) I have resigned from my highly sought after teaching position. (PID 536)

Participants described how their use of time changed due to the pandemic, particularly for crucial health behaviours like physical activity and alcohol consumption. Mixed effects were reported. Some participants had increased their physical activity, while others had decreased. Stay-at-home and social distancing measures meant more time with the family for some.

The increase in family time has been a massive positive. (PID 252)

I have stopped drinking alcohol to protect my health since the pandemic. This has supported my mental health. (PID 7)

While for others, these measures were isolating:

Impacts of isolation from working at home are underestimated and there is little attention paid to those people who actually already live by themselves and so don’t have family around them.(PID 19)

The impacts of these changes on mental health were described. While some participants had adopted healthier habits to protect their mental health, others found that healthy habits were falling by the wayside due to poor mental health.

I have suffered depression and anxiety in the past, and this has certainly taken a toll on my mental health (although compounded by relationship and work stress). I’ve had my first panic attack in a number of years earlier this week. There’s silver linings in everything. I’m getting more exercise, spending more time with my kids, and eating healthier, and I’ve enrolled in the (currently free) online THIS WAY UP course - some good reminders in Lesson 1. (PID 68)

### Disease outbreaks

A different theme related to the risk of school-based infections, outbreaks, and transmission of COVID-19. Some participants were concerned about self-infection. However, concerns about infecting others were more prevalent. Participants were worried that they would transmit COVID-19 to their students, family members, and other people in the community. This was particularly pronounced for participants who had vulnerable family members at home, with individuals questioning the safety of interaction with children and other adults all day and then going home where different social distancing rules applied. Several reported taking additional precautions to avoid potentially transmitting the infection to grandparents:

A town like mine, where many children are cared for by their grandparents or where they have less than ideal or hygienic homes due to social factors, COVID-19 would be devastating, and our little country hospital wouldn’t have a hope to cope with a community outbreak. I’m scared for my loved ones and my community. (PID 402)

Concerns were also expressed for children that were at greater risk due to underlying health conditions and other risk factors including being Aboriginal:

I feel sick at the thought that after a day in the classroom with young children I go home to my Aboriginal son who has diabetes and hypertension and I also have hypertension and a chronic medical condition but we don’t fit the profile for being ‘at risk’ due to our age. I come home from work and risk my own child’s life every day by the contacts with children and other staff I have no choice but to be in while at work. (PID 274)

These concerns were also extended to family members at home with health conditions such as Parkinson’s Disease or asthma.

My husband has Parkinson’s Disease and has had one bout of pneumonia this year, and my youngest (14yo) severe asthma and a weakened immune system due to two previous bouts of whooping cough (fully vaccinated), and he still gets croup on a regular basis. Concerns my exposure to adults and children at my workplace, who may be asymptomatic or parents who send sick kids to school, will place myself at high risk of infecting my own family. (PID 104)

### Working conditions

Participants reported facing substantial challenges related to changes to work conditions. These included increased workload, difficulty preparing lessons in multiple formats, insufficient access to cleaning supplies and personal protective equipment (PPE), and entitlement concerns related to sick leave and pay.

#### Increased workload

About one-third of participants reported increased workloads due to school opening status changes. This included the need to rapidly upskill themselves and students in remote education delivery formats while ensuring that students had access to the technology they needed or paper-based alternatives.

I have spent a lot of overtime in my own time creating and upskilling to be able to teach online only to feel like it has been a possible waste of time. (PID 220)

Another causal driver for increased workload was adapting lessons and materials for multiple delivery formats, including remote teaching during lockdowns, standard face-to-face instruction, and mixed delivery for students staying home while others were at school.

As a teacher, I’m currently feeling extremely let down and feeling as though I am being asked to do double if not triple the workload. I am feeling extremely overwhelmed and under pressure currently. To hear that it’s possible and a normal curriculum should be followed makes me feel very uneasy. I’m unsure how I’m supposed to do everything they’re asking me to do and follow a normal curriculum when parents have the choice to send their children or not. As a kindergarten teacher, it’s extremely difficult to teach the necessary concepts online. (PID 381)

Teachers also worried about their students’ ability to cope, which provided additional stress:

Students can’t cope with online learning as they don’t have the IT skills or the mental supports to keep going. (PID 191)

…worrying about kids with no devices…’ (PID 562)

Despite new cleaning and hygiene protocols, participants reported that their schools and classrooms were not regularly cleaned. They had insufficient access to cleaning services and supplies like face masks, hand sanitiser and soap.

How can the beach be unsafe but my school where the cleaning is NOT done as department says we didn’t even have hand sanitiser at all as for soap had to ask for donations. Playground was never cleaned. Why can I not go to my local park but kids can play with blocks and toys that haven’t been cleaned. Also, no social distancing at school at all. Just think we should be all just go free if school is safe.(PID 235)

We do not have adequate supplies of cleaning materials (at the end of last term, we were on the dregs of sanitiser). We also were short on cleaning staff which is likely to get worse. (PID 274)

Participants reported changes and impacts on their work entitlements and broader financial security as additional stressors. This included not having access to enough leave or flexibility and autonomy to alter their work patterns to protect their health and their families. This included teachers with medical conditions like asthma or family members with these conditions or being pregnant and unable to avoid exposure to high-risk environments. Further, the broader economic impacts of COVID-19 on families also affected teachers’ financial security, some of whom supported other family members whose income had been reduced.

Feel like I have no choice but to go to work. I have several medical conditions that leave me less prone to fight this virus. My boss said to come to work or you will have to take leave without pay. No other offer of working from a distance, she didn’t care. I wanted to use a face mask she said no I will scare the children. PID 567

## Discussion

This study assessed Australian schoolteachers’ perspectives on how they had been affected by the COVID-19 pandemic with data collected shortly after a critical policy shift (the sudden reopening of schools). Our findings suggest that public health policy caused substantial stress for teachers in Australia, including increased workload, and was detrimental to teachers’ working conditions and wellbeing. Policy implementation was a significant stressor, with participants feeling that teachers were subjected to different standards than the rest of the population and, therefore, felt that they were co-opted as ‘guinea pigs’ in the government’s public health response. For example, teachers were frustrated and angry that they were subjected to strict social isolation rules in their private lives yet were expected to teach in-person to large groups of children where social distancing and hygiene measures were not possible and when the transmission potential of children was poorly understood. These stressors were compounded by increased occupational stress due to increased workload, worsening working conditions, and lack of access to adequate cleaning supplies.

The abrupt opening of schools in April 2020 caused significant stress for Western Australian schoolteachers. In particular, participants felt that the expectation that teachers could quickly pivot between face-to-face and remote learning models and prepare multiple delivery modes was unrealistic, with negative consequences for wellbeing, job satisfaction, and morale. Teacher morale is a critical factor for job success and is influenced by personal, professional, and school-level factors (Gemeda & Tynjälä, 2015; Mackenzie, 2007). Findings from the current study suggest a broad negative impact on teacher morale caused by this sudden shift in policy across all three levels (Mackenzie, 2007). This is particularly alarming when considered against the backdrop of research that reported inordinately high levels of occupational stress and problematic levels of attrition and burnout in the teaching profession (e.g. García-Carmona et al., 2019; Prilleltensky et al., 2016; Skaalvik & Skaalvik, 2015). Additionally, the implementation of public health policy runs the risk of exacerbating already problematic levels of burnout, stress, and attrition among teachers, as well as issues of low morale and low sense of professional identity that have been documented among Australian teachers (Heffernan et al., 2019, 2022).

Similar to previous research, teachers noted a wellbeing decline at this early pandemic stage. This included physical and mental components such as changes in health behaviours and worsening mental health. This is consistent with the small body of research that has explored teachers’ experiences during the pandemic (Ozamiz-Etxebarria et al., 2021). For example, a longitudinal qualitative study with 71 British schoolteacher participants showed detrimental impacts on their wellbeing, primarily driven by increased workloads, increased job-related uncertainty, and negative perceptions of the profession (Kim et al., 2022). This study also demonstrated a worsening in these stressors from April 2020 to November 2020.

Only a few teachers commented positively on the government’s response to the pandemic. Our study represents a snapshot in time where we captured teacher sentiment in the immediate reaction to a sudden policy change that heavily impacted them specifically. Shortly after the announcement to reopen schools, likely in response to the backlash from the education community, broader COVID-19 restrictions were eased somewhat. The Australian COVID-19 pandemic experience has been relatively favourable, characterised by lower infection rates and proactive COVID-19 containment policies (Migone, 2020). Yet, even in 2022, with most of the population vaccinated, schools remain an epicentre of the COVID-19 pandemic, highlighting the critical link between public health policy and education systems.

## Future research/practice

Looking to the future, ways must be found to improve teachers’ working conditions and mitigate the detrimental impacts of public health policy on teachers. Previous research has also found that interventions can help to promote increased wellbeing (Hinze & Morton, 2017) and resilience (Fernandes et al., 2019) among teachers. Still, it is unclear how readily available they are. Further research is needed to understand how such programmes and interventions can be implemented in schools (Fernandes et al., 2019).

Our findings raise the question of what could have been done differently. Teachers are a resilient and adaptive group; however, it is clear that how the school reopening policy was implemented caused significant distress for teachers. Lessons learned from this experience and the stories shared by the teachers should be heeded and remembered for future public health and other crises. In the future, a more inclusive approach to policy development is likely to help (Berhrstock-Sherratt & Rizzolo, 2014). This can result in more feasible policies for teachers to implement and experience greater acceptance. Additionally, the approach could efficiently address some of the issues (e.g. lack of cleaning supplies and PPE) that would be relatively simple to fix but must be a top priority since they directly affect teachers’ health and cause a large amount of stress. Lessons learned about how interactions can be limited in schools, e.g. cancelling full assemblies, staggered lunch breaks, and zoned year groups, should also be documented and incorporated into social distancing protocols in schools.

There is increasing evidence regarding the positive and negative effects of school-based measures for containing COVID-19 (Isphording et al., 2021; Kratzer et al., 2022; Lee et al., 2020), as well as growing calls to strengthen education systems’ preparedness for future pandemics, other disasters, and overlapping disasters (Lennox et al., 2021). However, as our findings reinforce, policy implementation remains a critical determinant of their efficacy in strengthening the preparedness of education systems (Lennox et al., 2021; Vincent-Lancrin et al., 2022). Our findings also highlight the challenges that teachers face in implementing policy that they are not consulted on or given an opportunity to contribute to, even though it severely affects their profession and daily routines. Moving forward, inclusive policy development that occurs in partnership with teachers, the government, and other stakeholders, is required.

Consistent with previous research (Heffernan et al., 2019), our findings corroborate that there exists an underlying feeling amongst the education community that their profession is undervalued within Australian society. The reaction from educators uncovered by our study was strong, and there was a consistent sentiment that participants felt like guinea pigs and were subjected to unfair and experimental rules that jeopardised their health and wellbeing and their families. That teachers may feel undervalued in Australian society has been acknowledged as an issue before the pandemic (e.g. Mackenzie, 2007) and more recently (e.g. Heffernan et al., 2019). One mechanism that maintains perceptions of being undervalued is skewed media reporting on education in Australia that typically focusses more on negative than positive stories (Shine & Rogers, 2021).

## Strengths and limitations

The findings reported here are part of a more extensive online survey-based study in the data collection phase at the Western Australian premier’s announcement regarding reopening schools for term 2 in 2020. The survey was completed by 372 teachers, of which 98% were female, which is higher than estimates that suggest 78% of Australian teachers are female (Australian Institute for Teaching & School Leadership, 2018). Thus, a snapshot in time of an unprecedented event was captured. During a sudden policy-driven switch from remote to in-person teaching, data on teacher perspectives are unique and not routinely captured. Consequently, the survey at the time did not collect some school-specific variables that would be of great interest to the findings reported here. For example, no information on the type of educational setting (i.e. kindergarten, primary school, secondary school, and special needs), type of school (i.e. public vs private) or the location of the school (i.e. urban vs rural; low socioeconomic status vs high socioeconomic status) was collected.

Based on the comments, we suspect that kindergarten teachers and those teaching the early primary years would be more concerned regarding COVID-19 given difficulties in socially distancing children and basic hygiene practices given the young ages of these children and no mask wearing. These difficulties are also likely shared by some individuals teaching children with special needs depending on their needs. The type and location of the school may have shaped participants’ comments, particularly regarding the availability of cleaning products/services and personal protective equipment, which may be less available in public schools and those of low socioeconomic status. Difficulties with online learning are also likely to be more prevalent the younger the students are and for those with special needs. The student’s age is expected to be inversely related to how much parental support they would need in accessing the online learning resources. Furthermore, some teaching would not readily lend itself to online learning.

It must also be acknowledged that many comments about the government could be partially attributed to the general comments question at the end of the survey explicitly asking participants regarding ‘feelings about the government response’ amongst other aspects of the pandemic.

## Conclusions

The COVID-19 pandemic has caused immense disruption to educators and schools globally, including, but not limited to, widespread school closures, a switch to remote and mixed lesson delivery methods, and economic and social challenges (UNESCO, 2022). Our findings highlight the adverse consequences for teachers caused by the COVID-19 pandemic and public health policy. According to teachers, the pandemic increased their workloads and occupational stress and negatively impacted their wellbeing. Our findings also highlight teachers’ perceptions of inadequacies in terms of how policy was implemented; for example, the rapid pace at which teachers were expected to pivot to remote lesson deliveries was perceived to reflect policy makers’ misunderstanding and gross under-valuing of teachers’ roles. This conflict was compounded by the expectation that teachers continue to work in environments where they could not keep socially distanced, putting their own families and networks at risk and feeling like they were the guinea pigs for policy experimentation. These findings add to a chorus of previous researchers and advocates who have called for more feasible policy and greater acceptance of policy by having *everyone at the table* of education policy implementation (Berhrstock-Sherratt & Rizzolo, 2014).
